# Mapping young children’s executive function and motor skill profiles to emotional and behavioral problems: a latent profile analysis

**DOI:** 10.3389/fpsyg.2026.1798115

**Published:** 2026-06-24

**Authors:** Darren R. Hocking, Owen Yang, Charlotte Mcloghlin, Amiera Al Sidek, Kate E. Williams

**Affiliations:** 1 Institute for Health and Sport, Victoria University, Melbourne, VIC, Australia; 2 Developmental Neuromotor and Cognition Lab, School of Psychology and Public Health, La Trobe University, Melbourne, VIC, Australia; 3 School of Education and Tertiary Access, University of the Sunshine Coast, Sippy Downs, QLD, Australia

**Keywords:** executive function, internalizing and externalizing behavior, latent profile analysis, motor skill, parent report, preschool child

## Abstract

Although a close interdependence between motor coordination and executive functioning (EF) has been widely recognized in early childhood, we currently lack a systematic understanding of how intrinsic heterogeneity of motor and EF skill development conjointly affects children’s emotional and behavioral problems. Therefore, we used latent profile analysis (LPA) in typically developing preschoolers to (1) identify distinct constellations of motor and EF skills that characterize subgroups of children, (2) determine how motor and EF profile membership is associated with behavioral problems, and (3) identify the extent to which motor and EF profile membership is associated with behavioral difficulties and demographic characteristics (i.e., age, gender maternal education). Parents/guardians of 422 children aged 2 years 5 months to 5 years 9 months completed informant-based measures related to their child’s motor skills, EF, and internalizing/externalizing behavioral problems. The LPA revealed five distinct profiles: the “Competent” profile with average motor skills and EF (Profile 1: 38.9%); the “Poor EF” profile with average motor skills and below average EF (Profile 2: 21.1%); the “At-Risk” profile with weak motor skills and the poorest EF (Profile 3: 15.4%); the “High Motor” profile with strong motor skills and above average EF (Profile 4: 12.6%); and the “Highly Skilled” profile with above average motor skills and the highest EF (Profile 5: 12.1%). Compared to the Competent profile, children in the At-Risk profile had the highest odds of elevated internalizing problems but no elevation in externalizing problems, while children in the Poor EF profile had the highest odds of elevated externalizing problems but no relative elevation in internalizing problems. These findings could identify at-risk subgroups for early movement-based interventions that strengthen children’s motor and EF skills to reduce the frequency of behavioral problems and improve school readiness.

## Introduction

Early childhood is a key developmental period, in which cognitive and motor skills develop at a markedly rapid pace and provide the foundation for long-term success in other developmental domains including language, social skills and socio-emotional functioning (Gandotra et al., 2022; Kuzik et al., 2020). One example of an important cognitive skill is executive functioning (EF), which refers to a set of higher-order cognitive processes that enable control over goal-directed thoughts, emotions and action (Diamond, 2013). Another closely related key developmental domain is motor skills, which refers to movements of the body required for daily activities including balance and postural control, ballistic skills, and unimanual/bimanual coordination (Barnett et al., 2009; Roebers and Kauer, 2009). Although early childhood has been widely acknowledged as critical for motor, EF and psychosocial development, there has been a paucity of studies examining relations among these domains in preschool children. There is a growing body of evidence to suggest that motor difficulties expose a child to a cascade of secondary stressors, which subsequently lead to negative self-appraisals and poor psychosocial health (Erskine et al., 2024; Rigoli et al., 2012, 2017). In turn, problems in EF skills are strongly associated with later problem behaviors including externalizing/internalizing symptoms in preschoolers (Hughes et al., 2010; Hughes and Ensor, 2011). However, the extent to which both difficulties in motor skills and EF interact to impact these psychosocial issues in the preschool period remains poorly understood.

There is an emerging body of evidence highlighting a close association between motor coordination and EF skills in normative samples of preschool and school aged children (Oberer et al., 2017; Roebers and Kauer, 2009; Stöckel and Hughes, 2016), and in 5–7 year-old children with motor coordination impairments (Michel et al., 2011). Motor skills defined as body movements, can be broadly categorized into gross and fine motor skills. Gross motor skills involve the effective navigation through space using large muscles in the torso, arms, and legs, whereas fine motor skills refer to the coordination of small muscle movements in the fingers, hands, and wrists to manipulate objects efficiently (Haywood and Getchell, 2009). Regarding EF skills, it is generally accepted that these higher-order cognitive processes include *inhibition*, the ability to override or suppress dominant or automatic reactions to favor what is more appropriate or needed (Diamond, 2013; Friedman and Miyake, 2017); *cognitive flexibility*, the ability to shift attention between tasks, rules, or perspectives in response to changing demands (Diamond, 2013; Miyake et al., 2000); and *working memory updating*, the ability to temporarily hold and manipulate information in mind over short periods of time to guide behavior relevant to a current task (Miyake et al., 2000). It has been proposed that shared brain structures (prefrontal cortex and cerebellum) subserving motor skills and EF show a rather protracted developmental trajectory, are associated with cognitive and motor problems in developmental disorders, and suggest a common underlying mechanism (i.e., cerebro-cerebellar connectivity) (Diamond, 2000). Yet, the evidence from meta-analyses have found small effect sizes for relationships between motor skills and EF in typically developing (TD) children aged 3–12-years-old (Gandotra et al., 2022), and in those aged 4–16-years-old (van der Fels et al., 2015). The widely discrepant findings in the motor-EF link during childhood and adolescence may be the result of methodological differences (i.e., selection of a wide range of motor and/or EF tasks) and/or heterogeneity of motor and EF abilities across these sensitive periods of development.

In addition to the motor-EF link, it has been suggested that motor coordination difficulties reduce opportunities for social interaction during active play, which in turn could confer risk for poorer psychosocial health in young children (Li et al., 2025). Importantly, children with motor coordination difficulties consistently display a range of emotional and behavioral problems (Crane et al., 2017; Lingam et al., 2012; Skinner and Piek, 2001). One aspect that has been linked to motor skills is internalizing problem behaviors, which refers to psychological or emotional issues that are directed inward, often manifesting as anxiety, depression, social withdrawal, and somatic complaints (Eaton et al., 2013; Kovacs and Devlin, 1998). Although some studies in TD preschoolers have found a direct association between motor skills and internalizing problems (Katagiri et al., 2021, Piek et al., 2008; Rodriguez et al., 2019), the literature examining this association in children under 7 years of age remains inconsistent (Hirata et al., 2021; King-Dowling et al., 2015; Mancini et al., 2018). For instance, Rodriguez et al. (2019) showed that preschool children with motor difficulties had more internalizing problems (anxiety/depression, and withdrawn behaviors above clinical threshold), whereas King-Dowling et al. (2015) reported no significant group differences in overall internalizing problems in preschoolers with motor difficulties relative to their TD peers. Some possible explanations for these discrepant findings might relate to differences in sample size between these studies [*n* = 582 in Rodiguez et al. (2019) versus *n* = 214 in King-Dowling et al. (2015)], or potential unidentified mediators or moderators of the association between motor skills and internalizing problem behaviors (e.g., EF skills, social skills).

In addition to the important role of motor proficiency, recent conceptual models propose that EF impairments are a transdiagnostic risk factor for several social/behavioral issues including conduct problems, anxiety and aggression (Lynch et al., 2021; Zelazo and Carlson, 2020). Consistent with this, a recent meta-analysis provided evidence for longitudinal associations between EFs and a broad range of internalizing problems, but not with subsequent behavioral issues related to anxiety in children and adolescents aged 3–17 years old (Yang et al., 2022). In TD samples, evidence from longitudinal studies show robust and specific associations between individual differences in EF and later emotional difficulties in children aged 4–6 years of age (Hughes and Ensor, 2011). One explanation for the association between poorer EF and internalizing problems in children is that inhibitory control (one of the core EFs) is important for controlling emotions and impulsive behaviors (Carlson et al., 2002; Hughes et al., 2000). Although this research has advanced our understanding of links between EF and internalizing problems, the extent to which motor coordination difficulties and EFs interact to directly impact these psychosocial issues in early childhood remains unclear.

Importantly, poor motor coordination and EF difficulties are related to developmental problems in externalizing behaviors as well, which refer to children’s negative or maladaptive behaviors directed toward one’s environment (e.g., inattention/hyperactivity, oppositional defiance, bullying and conduct problems; Schwartz, 2011). In relation to problem behaviors, one meta-analysis of 22 studies found that preschool EF, especially inhibition, is significantly associated with externalizing symptoms in children aged 3–6 years with externalizing problems (i.e., clinical diagnosis or symptoms of ADHD, conduct disorder, oppositional defiant disorder; Schoemaker et al., 2013). In normative TD samples, several longitudinal studies show robust and specific associations between individual differences in EF and teacher-rated problem behaviors (e.g., emotional symptoms, hyperactivity, conduct/peer problems) in children aged 2–4 years (Hughes et al., 2010) and in older children 4–6 years of age during transition to school (Hughes and Ensor, 2011). The meta-analysis by Yang et al. (2022) also showed that greater EF abilities were prospectively associated with less externalizing problem behaviors including fewer conduct problems, and less attention deficit/hyperactivity disorder (ADHD) and oppositional defiant disorder symptoms in children and adolescents (aged 3–18 years). One explanation is that children with poor EF are likely to experience more difficulty in controlling impulses and regulating emotional responses, which in turn could result in an increased risk of acting out externally with negative or maladaptive behaviors.

Currently, however, the extant research examining the link between poor motor skills and externalizing problems is scarce, with the majority of these studies including children with or at-risk for Developmental Coordination Disorder (DCD: James et al., 2022; King-Dowling et al., 2015; Rodriguez et al., 2019; Schoemaker et al., 2024; Wagner et al., 2012). DCD is a neurodevelopmental condition characterized by poor motor skills that significantly impact daily life, in the absence of genetic, neurological, or global developmental delay (Blank et al., 2019; Lingam et al., 2010). These motor difficulties interfere with a child’s ability to perform everyday tasks and can significantly impact self-care, recreational activities, and social participation (Asonitou et al., 2012). More recently, a retrospective study by Schoemaker et al. (2024) examined clinical records from 93 children with a clinical diagnosis of DCD (aged 5–12 years) and found that two-thirds of the children displayed emotional or externalizing behavioral problems according to both parent and teacher reports. Importantly, the most pronounced difficulties were in a subtype of DCD with generalized gross and fine motor impairments (Schoemaker et al., 2024). However, it remains unclear to what extent similar links between motor coordination and behavioral problems exist in TD children with poor motor skills or those at-risk for DCD in the preschool period. This is important because children with difficulties in motor skills often show reduced participation in active play, and experience failure in everyday activities, which can increase risk for peer exclusion, frustration and negative emotionality (Pimenta et al., 2023). Therefore, the identification of an at-risk motor-EF profile in young TD children could inform early interventions to reduce cascading effects on social-emotional development and may support the identification of children at-risk of future developmental disorders.

The extent literature on the relations among motor skills, EF, and problem behaviors in young children reveals several issues that need to be addressed. First, several of the previous studies employed performance-based tasks of EF or motor skills in older children. Although performance-based tasks may give a detailed account of the child’s motor or EF capacities under highly standardized conditions, they do not correspond to the multifaceted and dynamic nature of real-world situations based on observations of the child’s behavior in daily situations. Indeed, it has been increasingly recognized that performance-based measures of EF may lack ecological validity in capturing the multilevel nature of goal-directed behavior in children (Doebel, 2020; Perone et al., 2021). Second, there has been a relative dearth of research examining associations between motor-EF profiles and problem behaviors in young preschool children (i.e., 3–5 years of age), despite the importance of early childhood for development of motor skills, EF and psychosocial development. Finally, the majority of studies have relied on variable-centered approaches, such as correlational analysis and regression, which assume population homogeneity and are limited in their ability to capture the variability in motor and EF development among preschool children. By contrast, person-centered methods like latent profile analysis (LPA) offer a more nuanced perspective by identifying subgroups of children who share similar patterns of functioning. This approach makes it possible to uncover distinct combinations of motor and EF skills, hence providing deeper insight into diverse developmental pathways while addressing the oversimplification inherent in aggregated data (Bergman and Magnusson, 1997; Collins and Lanza, 2013).

To date, only a few studies have adopted a person-oriented approach and addressed the heterogeneity of motor performance or EF abilities in relation to other problem behaviors in preschool children. Using LPA, Houwen et al. (2019) used a sample of 119, 3–4-year-olds to identify three profile groups of preschool children based on different aspects of EF (both performance-based and informant reports) and assessed verbal ability and performance-based motor skills: an At-Risk group with below average motor, EF, and verbal ability (6.7% of sample); a group with average motor, below average verbal, and above average EF (43.3%); and a group with average motor, above average verbal, and elevated parent-reported EF problems but average performance-based EF skills (50%). Houwen et al. (2019) found that the At-Risk subgroup of children and those in the final profile with elevated parent-reported EF problems were more likely have parent-reported ADHD symptomatology and be clinically at-risk of motor coordination difficulties; however, there were no differences in age, gender, language problems and SES across the profiles. Using behavioral measures of self-regulation and performance-based measures of EF with 206, 4–5-year-old socioeconomically disadvantaged preschoolers, Williams and Bentley (2021) identified both low (35%) and high (65%) self-regulation/EF profile groups, with the low self-regulation/EF profile demonstrating a greater likelihood of poorer teacher reports of social skills and increased problem behaviors. In addition, the children in the high self-regulation/EF subgroup demonstrated better visual-motor integration skills, supporting the notion that motor and cognitive skills codevelop in preschoolers (Williams and Bentley, 2021). Regarding sociodemographic characteristics, the higher skilled profile was characterized by a greater ratio of girls but there were no differences across profiles for parental education. Given the overlap of motor skills and EF in this age range, and links between both domains and internalizing/externalizing problem behaviors in children with or at-risk for DCD, further research that examines them together in the preschool period is warranted.

### The current study

Based on these considerations, the primary aim of the current study is to examine whether distinct subgroups can be identified based on constellations of behavior across parent-reported motor skills and everyday EF in preschool aged TD children. The secondary aim is to elucidate how children in the identified profile memberships differ on both internalizing and externalizing problem behaviors. The final aim is to characterize the identified motor-EF profiles with regard to socio-demographic characteristics including age, gender, and maternal education, and whether these factors are disproportionately represented across subgroups. Given the exploratory nature of the study, it is not possible to make a specific prediction regarding the distinct response patterns of motor and EF skills and the number of possible subgroup profiles. However, on the basis of previous studies focusing on internalizing or externalizing problem behaviors in relation to motor or EF skills (Houwen et al., 2019; Williams and Bentley, 2021), it is hypothesized that at least one “At-Risk” profile group with both low motor skills and elevated EF difficulties will be identified. In addition, it is also expected that the higher skilled profile will have an increased likelihood of being female and having lower behavioral problems when compared to an average or “Competent” referent group (Williams and Bentley, 2021). However, the “At-Risk” profile will be characterized by higher ratios of children with increased internalizing and externalizing behavioral problems, but no specific predictions will be made regarding age related differences across profiles based on mixed findings in the literature.

## Materials and methods

### Participants

The final sample comprised 422 children (*M*_*age*_ = 3.93 years, SD_*age*_ = 0.89, range 30–69 months), with an even distribution of males and females (48.8% vs. 51.2%). Participants were recruited through the crowdsourcing platform Prolific^1^, an online recruitment tool that carefully checks for authenticity by conducting identity checks and verifying IP addresses. To be eligible, parents or guardians were required to be 18 years or older and have a typically developing child aged between 2 years 6 months and 5 years 11 months. As the survey was administered exclusively in English, proficiency in English was necessary to ensure accurate comprehension and completion of the survey and participants were required to live in an English-speaking country (Australia, USA, UK, or Canada). Children with severe motor dysfunction (e.g., cerebral palsy) or a diagnosed neurodevelopmental disorder (e.g., autism spectrum disorder, developmental coordination disorder) were excluded. There were no children with a formal diagnosis of Developmental Coordination Disorder (DCD), however 56.2% were identified as being at-risk for motor coordination difficulties, scoring in the probable DCD range (15–67 for boys and 15–68 for girls) on the Little Developmental Coordination Disorder Questionnaire – Canadian (Little DCDQ-CA; Wilson et al., 2009). Maternal education was used to determine family SES and was classified into three subgroups: high school or below (15.4%), diploma or vocational certificate (15.9%), and bachelor’s degree or higher (68.7%). Ethics approval for this study was obtained from the La Trobe Human Research Ethics Committee (HEC 20262) and the Victoria University Human Research Ethics Committee (HRE24-062).

### Measures

*The Little Developmental Coordination Disorder Questionnaire – Canadian* (Little DCDQ-CA; Wilson et al., 2009). The Little DCDQ-CA is a brief 15 item parent-report questionnaire designed to measure motor coordination difficulties in young children aged 3–5 years old. The scale is separated into gross and fine motor skills and consists of a series of statements rated on a five-point Likert scale ranging from Not at all like your child (1) to Extremely like your child (5). The maximum total score is 30 for gross motor skills and 45 for fine motor skills, with higher scores indicating better motor coordination. The maximum total score is 75 with high scores between 68–75 for boys and 69–75 for girls indicating fewer difficulties with motor skills (“probably not DCD”). Scores between 15–67 for boys and 15–68 for girls indicate a higher risk of probable DCD or that they are “Suspect for DCD.” The Little DCDQ-CA has been shown to have excellent internal reliability (Cronbach’s α = 0.94) and test-retest reliability (ICC = 0.96) (Wilson et al., 2014). The scale has also been reported as demonstrating high concurrent validity (Wilson et al., 2014) and good criterion validity as a screening tool (Hudson and Willoughby, 2021). Internal reliability estimates for all measures in the current study are shown in Table 1 in the Results section.

**TABLE 1:** Descriptive statistics and bivariate correlations.

Variable	*M*	SD	α	1	2	3	4	5	6	7	8	9
1. Female	–	–	–	–	−0.03	0.13**	0.21***	0.00	−0.03	−0.03	0.01	−0.08
2. Age (m)	46.76	10.65	–	–	–	0.13**	0.24***	0.05	0.04	0.00	−0.02	−0.01
3. Fine motor skills	38.99	5.68	0.83	–	–	–	0.74***	−0.23***	−0.29***	−0.25***	−0.29***	−0.17**
4. Gross motor skills	25.42	4.4	0.87	–	–	–	–	−0.20***	−0.24***	−0.27***	−0.22***	−0.20***
5. Inhibition/self-control	54.96	12.13	0.93	–	–	–	–	–	0.84***	0.82***	0.44***	0.76***
6. Flexibility	54.31	12.04	0.92	–	–	–	–	–	–	0.71***	0.58***	0.58***
7. Emergent meta-cognition	55.21	13.19	0.94	–	–	–	–	–	–	–	0.43***	0.70***
8. Internalizing problems	3.82	2.87	0.68	–	–	–	–	–	–	–	–	0.41**
9. Externalizing problems	6.4	3.82	0.81	–	–	–	–	–	–	–	–	–

*n* = 422. *M*, mean; SD, standard deviation; α, Cronbach’s α.

**P < 0.01.

***P < 0.001.

*Behavior Rating Inventory of Executive Function – Preschool Version* (BRIEF-P; Gioia et al., 2003). The BRIEF-P is a 63-item parent-report questionnaire that assesses several domains of everyday executive functioning. Parents rate a series of statements on a three-point Likert scale (Never, Sometimes, Often), indicating how often the behavior has been a problem in the last 6 months. There are five clinical scales (Inhibit, Shift, Emotional Control, Working Memory, and Plan/Organize) and three composite indexes: Inhibitory Self-Control comprised of the inhibit and emotional control clinical scales; Flexibility comprised of the shift and emotional control clinical scales; and Emergent Metacognition comprised of the working memory and plan/organize clinical scales, and a Global Executive Composite (GEC) score, which is derived from a sum of the clinical scales. For the purposes of the current study, only the composite indexes were used in the analysis. Clinical scales and composite index scores are converted to T scores based on the child’s age and sex (*M* = 50, SD = 10). The recommended cutoff for executive dysfunction and potential clinical significance is a T score of 65 with higher scores indicating greater impairment in executive functioning. The BRIEF-P has been shown to have good internal reliability (Cronbach’s α = 0.80–0.90; Gioia et al., 2003), and excellent test-retest reliability for all composite indexes and the GEC score (*r* > 0.80; Sherman and Brooks, 2010). The parent report measure has also demonstrated good convergent validity (Ebrahimi et al., 2015; Skogan et al., 2015).

Strengths and Difficulties Questionnaire (SDQ; Goodman, 1997): The SDQ is a 25-item parent report questionnaire used to assess emotional and behavioral problems in children aged 2–17 years. The measure includes five subscales: Emotional Problems, Conduct Problems, Hyperactivity/Inattention, Peer Relationship Problems, and Prosocial Behavior. Each item is rated on a three-point Likert scale (0 = Not True, 1 = Somewhat True, 2 = Certainly True). For the current study, only the emotional symptoms and peer problems subscales were used to assess internalizing symptoms, while conduct problems and hyperactivity/inattention subscales were used to evaluate externalizing symptoms (Goodman et al., 2010). Higher scores on these composites indicate greater levels of internalizing or externalizing problem behaviors. The internalizing symptoms composite has demonstrated good internal consistency (Cronbach’s α = 0.64; Laugen et al., 2024), and moderate test–retest reliability over a 4–6-month period (*r* = 0.60; Goodman, 2001; Downs et al., 2012). There is also evidence for convergent validity with established measures of emotional behavioral problems such as the Child Behavior Checklist (CBCL; Goodman, 2001).

### Procedure

Participants completed the online survey via Qualtrics, which began with a Participant Information Statement outlining the purpose of the study, the nature of participation, and an Informed Consent Declaration detailing participants’ rights. After providing implied consent with the commencement of the survey, parents completed demographic questions about their child, including gender and chronological age (in years and months). Next, participants completed a battery of questionnaires, including the Little DCDQ-CA, BRIEF-P, SDQ, and several other measures from a larger study on the impact of motor skills on the cognitive and motor landscape in preschool children. The questionnaires took approximately 30 min to complete and were presented in a fixed order for all participants. Parents were able to withdraw their participation at any point during the study, and their responses were omitted from any subsequent analyses. Participants were reimbursed the equivalent of AUD$16 for their time.

### Data analysis

All analyses were conducted using R (R Core Team, 2025; Version 4.5.2) and Mplus (Muthén and Muthén, 2025; Version 9). Latent Profile Analysis (LPA) was used to identify meaningful subgroups based on fine and gross motor scores from the DCDQ-CA, and the inhibitory self-control, flexibility and emergent metacognition indexes from the BRIEF-P. To facilitate comparison across measures, z-standardized values were adopted. Prior to running the LPA, all continuous variables were screened for univariate outliers and values exceeding ±3.29 SD from the mean were adjusted (“winsorized”) to 3.29 SD from the mean (Tabachnick and Fidell, 2019). This approach minimizes the risk of extreme values artificially influencing profile formation, which can lead to spurious latent profiles (Spurk et al., 2020). A total of seven scores across the five variables were adjusted in this manner. Multivariate outliers were then assessed using Mahalanobis distance (D^2^), with a critical value of 20.52 and seven outliers were identified and subsequently removed from the sample. Normality was not evaluated prior to the LPA, as the assumption of normality is implied within each latent profile rather than across the overall sample (Bauer, 2022), and when samples include multiple subpopulations, the assumption of a normal distribution for the whole sample is not required.

To estimate the latent profiles, models specifying 2–6 classes were evaluated using Mplus with the robust maximum likelihood estimator (MLR). Given the strong dependence between fine and gross motor skills and among BRIEF-P indexes, residual covariances were relaxed to account for local dependence (Marsh et al., 2009; Morin et al., 2016). Specifically, covariances were freed between the variables and variances were allowed to be freely estimated across classes. To prevent the models from converging on local maxima, a high number of random starts were specified and this included 3,000 initial stage starts and 500 final stage optimizations with 200 iterations (Hipp and Bauer, 2006).

The optimal number of profiles was determined using a combination of statistical fit indices, classification accuracy, and theoretical interpretability (Nylund-Gibson and Choi, 2018). The information criteria evaluated included the Akaike Information Criterion (AIC) and the sample-size adjusted BIC (aBIC). Given the moderate sample size, aBIC was prioritized as the primary information criterion given recommendations for superior accuracy in class enumeration for smaller samples (Nylund et al., 2007). The Bootstrapped Likelihood Ratio Test (BLRT) was used to compare nested models, with a significant *p*-value (<0.05) indicating that the k-class model provides a significantly better fit than the k-1-class model. Entropy was used to evaluate classification quality, with values closer to 1.0 representing clearer delineation between classes. Finally, parsimony and clinical utility were considered, ensuring no emergent class was too small to warrant meaningful interpretation (Lubke and Neale, 2006).

Following class enumeration, we examined whether demographic covariates (age, binary sex and maternal education), and concurrent behavioral problems (SDQ internalizing and externalizing symptoms) predicted the likelihood of profile membership. To prevent these covariates from causing problematic shifts in latent class formation (Asparouhov and Muthén, 2014), we used a bias-adjusted three-step approach. Rather than relying on strict modal class assignment, this method applies classification weights derived from the final unconditional latent profile model. Thus, we conducted a multinomial logistic regression with Bose-Chaudhuri-Hocquenghem (BCH) weighting to account for classification uncertainty and quantification error. The referent group for all multinomial regression analyses was the “Competent” or average profile, to determine the characteristics that differentiated higher skilled profiles from the lower skilled profiles.

## Results

### Descriptive analysis

Table 1 presents descriptive statistics, internal reliability estimates, and bivariate correlations for all variables of interest. Overall, girls had better motor skills and older children showed higher scores on fine and gross motor skills (significant weak positive correlations) but there were no gender or age correlations with EF or behavior problems. Poorer EF skills were strongly positively correlated with greater levels of internalizing and externalizing symptoms, while there were generally weak negative correlations between motor coordination and both EF skills and behavioral problems. Internal consistency across the scales varied from acceptable to excellent (α ranging from 0.81 to 0.94) with the exception of externalizing problems (α = 0.68).

### Latent profile analysis

Table 2 provides a summary of the model fit indices derived from the LPA. Model solutions ranging from two to six profiles were evaluated to determine the optimal representation of latent subgroups. Both the AIC and SSA-BIC values continuously decreased with the addition of each profile, and the BLRT remained statistically significant (*p* < 0.001) across all tested solutions. Although the four-profile solution demonstrated adequate fit, it was deemed suboptimal as it merely split an existing high-skill group without adding meaningful qualitative distinctions. In contrast, the five-profile solution revealed a distinct structural change in the latent groups that provided a more theoretically meaningful representation of the data. Furthermore, while the six-profile solution showed lower AIC and SSA-BIC values alongside higher classification accuracy (Entropy = 0.84), it produced a smallest class size comprising only 3.1% (*n* = 13) of the sample. This class size is computationally insufficient and increases the risk of over-extraction. Consequently, the five-profile solution (Model 4) was retained as the optimal model. Although its entropy value of 0.77 falls slightly below the ideal target of 0.80, it remains acceptable and defensible within the field, particularly given that it maintains a robust smallest class size of 12.1% (*n* = 51) and provides the most qualitatively distinct and interpretable representation of the sample.

**TABLE 2:** Absolute and relative model fit indices and entropy values for each latent profile analysis (LPA) solution (*N* = 422).

Model	*k*	AIC	SSA-BIC	BLRT - *p*	Entropy	The smallest class size *n* (%)
1	2	4433.08	4454.87	<0.001	0.71	138 (32.7%)
2	3	4258.46	4289.84	<0.001	0.83	76 (18.0%)
3	4	4214.6	4255.57	<0.001	0.84	53 (12.6%)
4	5	4191.59	4242.14	<0.001	0.77	51 (12.1%)
5	6	4166.75	4226.9	<0.001	0.84	13 (3.1%)

*k*, number of profiles; AIC, Akaike’s Information Criterion; SSA-BIC, sample-size adjusted BIC; BLRT, Bootstrap Likelihood Ratio Test.

### Motor skills and executive functioning

**FIGURE 1:**
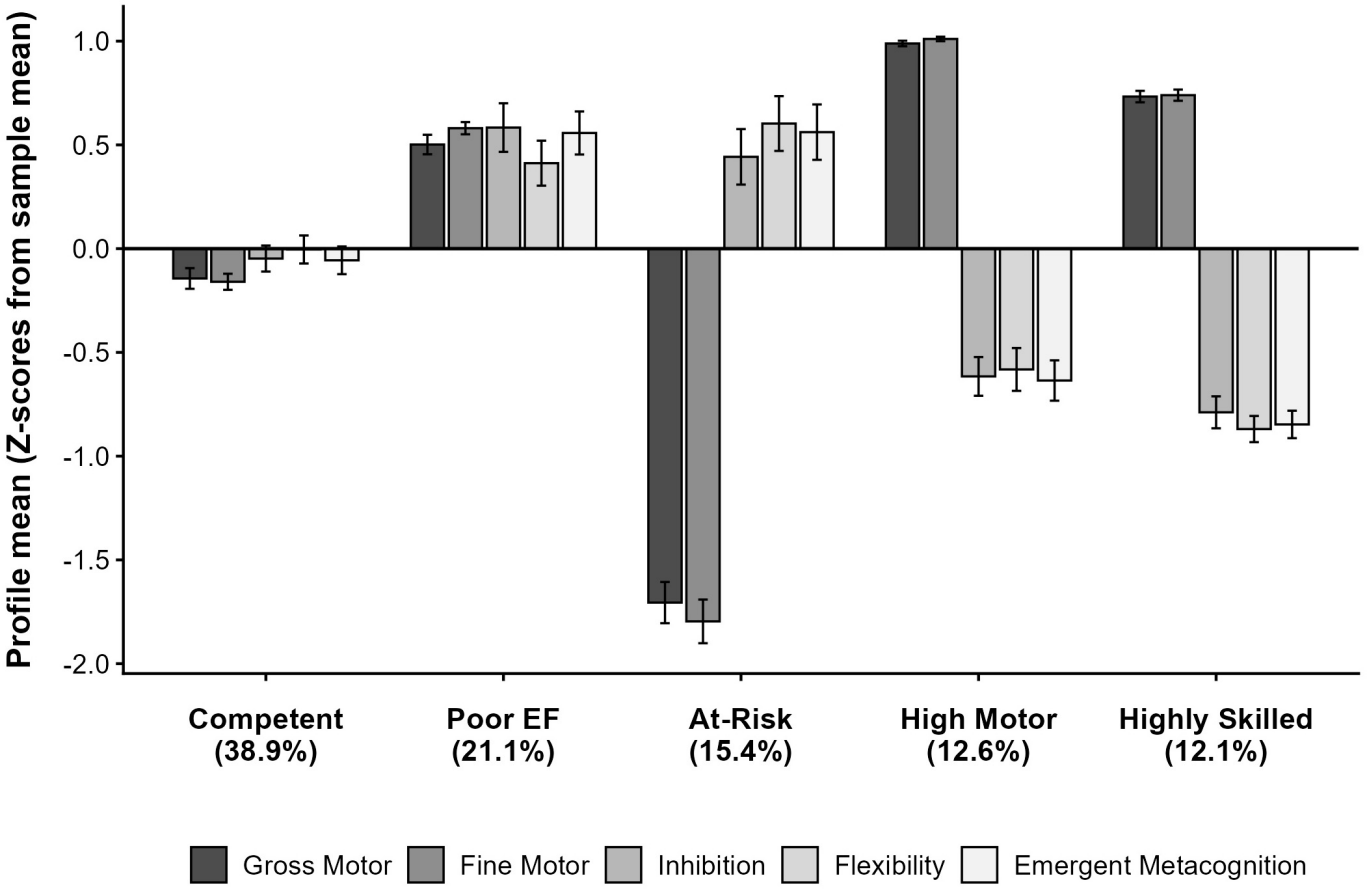
Latent profile plot of motor skills and executive functioning variables across profile membership (*N* = 422). Profiles are labeled based on their key functional characteristics. Bars represent z-scored means. Error bars indicate standard errors. Higher scores on BRIEF-P indexes (Inhibitory self-control, Flexibility, Emergent Metacognition) reflect more problems or poorer skills.

Figure 1 shows the distribution of the z-standardized scores for motor skills and BRIEF-P indexes across the 5 profiles, and descriptive statistics are reported in Table 3. Overall, Profile 1 was the most prevalent, comprising 38.9% of the sample (*n* = 164), and was defined by motor and EF skills that clustered closely around the sample average. We call Profile 1, the Competent profile. Profile 2 with 21.1% of the sample (*n* = 89) was characterized by average motor skills but below average skills across the three EF indexes. We call Profile 2, the Poor EF profile. Profile 3 (15.4%, *n* = 65) demonstrated the weakest fine and gross motor skills and the poorest EF skills of all profiles. We call Profile 3 the At-Risk profile. Profile 4 with 12.6% of the sample (*n* = 53) was defined by the highest overall fine and gross motor scores alongside above average executive functioning. We call Profile 4 the High Motor profile. Finally, Profile 5 as the smallest profile (12.1%, *n* = 51) was characterized by strong, above-average motor skills and highest of all profiles in EF skills. We call Profile 5 the Highly Skilled profile. Note that the labels selected for each profile reflect relative differences within the current sample and should not be interpreted as normative classifications.

**TABLE 3:** Descriptive statistics for motor skills and executive functioning across profile membership (*N* = 422).

	Profile 1 “Competent” *n* = 164, 38.9% *M* (SD)	Profile 2 “Poor EF” *n* = 89, 21.1% *M* (SD)	Profile 3 “At-Risk” *n* = 65, 15.4% *M* (SD)	Profile 4 “High Motor” *n* = 53, 12.6% *M* (SD)	Profile 5 “Highly Skilled” *n* = 51, 12.1% *M* (SD)
Motor skills					
Fine motor	38.09 (2.82)	42.29 (1.58)	28.78 (4.82)	44.74 (0.45)	43.20 (1.10)
Gross motor	24.79 (2.81)	27.63 (1.95)	17.91 (3.53)	29.77 (0.42)	28.65 (0.87)
Executive functioning					
Inhibition/self-control	54.38 (9.73)	62.03 (13.39)	60.32 (13.08)	47.49 (8.24)	45.39 (6.67)
Flexibility	54.26 (10.41)	59.27 (12.31)	61.57 (12.81)	47.30 (9.04)	43.84 (5.42)
Emergent metacognition	54.47 (11.26)	62.56 (12.90)	62.62 (14.19)	46.83 (9.33)	44.04 (6.23)

*M*, mean; SD, standard deviation. Higher scores on executive function measures reflect more problems or poorer skills.

### Characteristics of profile membership

The weighted multinomial logistic regression using the BCH method was conducted using the Competent profile (average motor and EF skills) as the reference group (see Table 4). This analysis revealed that the Poor EF profile had an increased ratio of children with higher SDQ externalizing scores than the Competent profile (OR = 1.20, 95% CI [1.12, 1.29], *p* < 0.001). The Poor EF profile did not show any differences in ratio of children in any of the demographic variables or SDQ internalizing problems. In contrast, the At-Risk profile was characterized by an increased odds of being younger age (OR = 0.70, 95% CI [0.51, 0.97], *p* = 0.033), being male (OR for females = 0.54, 95% CI [0.31, 0.97], *p* = 0.037), and higher in SDQ internalizing problems (OR = 1.15, 95% CI [1.04, 1.27], *p* = 0.006).

**TABLE 4:** Multinomial logistic regression predicting latent profile membership (*N* = 422).

Predictor	B	SE	OR	95% CI	*P*
Profile 2: Poor EF					
Age	0.17	0.138	1.18	[0.90, 1.55]	0.221
Gender (girl)	0.273	0.242	1.31	[0.82, 2.11]	0.259
Maternal education	−0.379	0.256	0.68	[0.41, 1.13]	0.139
SDQ internalizing	−0.085	0.046	0.92	[0.84, 1.01]	0.065
SDQ externalizing	0.183	0.035	1.2	[1.12, 1.29]	<0.001**
Profile 3: At-Risk					
Age	−0.355	0.167	0.7	[0.51, 0.97]	0.033*
Gender (girl)	−0.608	0.292	0.54	[0.31, 0.97]	0.037*
Maternal education	0.013	0.313	1.01	[0.55, 1.87]	0.968
SDQ internalizing	0.14	0.051	1.15	[1.04, 1.27]	0.006**
SDQ externalizing	0.078	0.042	1.08	[1.00, 1.17]	0.063
Profile 4: High Motor					
Age	0.598	0.185	1.82	[1.27, 2.62]	0.001**
Gender (girl)	0.544	0.326	1.72	[0.91, 3.26]	0.095
Maternal education	−0.672	0.336	0.51	[0.26, 0.99]	0.046*
SDQ internalizing	−0.17	0.072	0.84	[0.73, 0.97]	0.018*
SDQ externalizing	−0.108	0.054	0.9	[0.81, 1.00]	0.044*
Profile 5: Highly Skilled					
Age	0.175	0.173	1.19	[0.85, 1.67]	0.312
Gender (girl)	0.715	0.314	2.04	[1.10, 3.79]	0.023*
Maternal education	−0.068	0.348	0.93	[0.47, 1.85]	0.845
SDQ internalizing	−0.227	0.074	0.8	[0.69, 0.92]	0.002**
SDQ externalizing	−0.182	0.056	0.83	[0.75, 0.93]	0.001**

Profile 1 (“Competent”) served as the reference group for all comparisons. B, unstandardized coefficient; SE, standard error; OR, odds ratio; CI, confidence interval. The BCH step-3 method was applied to account for classification uncertainty in latent profile membership. Gender is coded as 1, boy; 2, girl (boy is the reference category).

*p < 0.05.

**p < 0.01.

For the profiles characterized by above average motor skills, the High Motor profile had significantly higher odds of being older (OR = 1.82, 95% CI [1.27, 2.62], *p* = 0.001), with higher maternal education (OR = 0.51, 95% CI [0.26, 0.99], *p* = 0.046), as well as lower SDQ internalizing (OR = 0.84, 95% CI [0.73, 0.97], *p* = 0.018) and externalizing symptoms (OR = 0.90, 95% CI [0.81, 1.00], *p* = 0.044). The profile marked by strong motor skills and the highest EF skills (Highly Skilled) was characterized by a higher ratio of girls (OR = 2.04, 95% CI [1.10, 3.79], *p* = 0.023), as well as less internalizing (OR = 0.80, 95% CI [0.69, 0.92], *p* = 0.002) and externalizing problem behaviors compared to the Competent profile (OR = 0.83, 95% CI [0.75, 0.93], *p* = 0.001).

## Discussion

The primary aim of the current study was to apply a person-centered approach to identify distinct profiles of motor skills and executive functioning (EF) in typically developing preschool-aged children. A secondary aim was to examine whether these motor-EF profiles varied in ratios of children with internalizing and externalizing problem behaviors, and how children in each profile membership differed across demographic factors including age, sex, and maternal education. To date, the existing studies have predominately focused on variable-centered approaches rather than exploring constellations of behaviors that characterize subgroups of children. Using a person-centered approach, five distinct profiles of preschool aged children with qualitatively distinct patterns of motor skills and everyday EF (i.e., higher or lower skills compared to the overall sample mean) were identified, and as hypothesized, an *At-Risk* profile group emerged with the weakest fine and gross motor skills and poorest EF abilities, comprising 15.4% of the sample. Consistent with expectations, the *At-Risk* profile were characterized by higher ratios of children with increased internalizing problem behaviors, and a higher odds of being male than the *competent* (“average”) profile. One novel finding is that our *Poor EF* profile included a higher ratio of children with elevated levels of externalizing problems but this was not the case in the *At-Risk* subgroup. These findings build upon previous evidence using performance-based assessments (Houwen et al., 2019; Williams and Bentley, 2021), to demonstrate the utility of a person-centered approach in young TD children with implications for early identification of children at-risk of poor emotional and behavioral wellbeing.

In line with the first objective, the current findings revealed five qualitatively distinct constellations of motor and EF skills among TD preschool children. In descending order of prevalence these were: motor and EF skills clustering close to the overall sample mean (Profile 1 – *Competent*: 38.9%); above average motor skills and below average EF (second lowest among profiles; Profile 2 – *Poor EF*: 21.1%); weak fine and gross motor skills (second lowest of all profiles) and poorest EF skills of all profiles (Profile 3 – *At-Risk*: 15.4%); highest fine and gross motor skills and average EF (Profile 4 – *High Motor*: 12.6%); and strong, above-average motor skills and highest of all profiles in EF skills (Profile 5 – *Highly Skilled*: 12.1%). Regarding the EF domains, the *Poor EF* profile consistently reached the “mildly elevated” threshold for EF indexes, whereas the *At-Risk* profile was identified as the weakest across all BRIEF-P indexes, with scores within the “clinically significant” range. Conversely, the *Competent* and *High Motor* profiles had children who scored within average to above average ranges across the BRIEF-P indexes, while the *Highly Skilled* profile demonstrated the strongest EF skills.

Of interest, is the extent to which motor skills together with EF clustered within profiles and distinguished our most *At-Risk* profile from other profiles. While our *At-Risk* subgroup had the lowest overall motor skills across all profiles, all other profiles included average or above average motor skills, and were distinguished primarily in terms of EF abilities varying from average to clinically elevated scores. The two profiles with the highest motor skills also had the strongest EF skills, suggesting a clustering together of these developmental areas for these two profiles (*High Motor* and *Highly Skilled* profiles). However, for our *At-Risk* and *Poor EF* clusters characterized by the poorest EF scores, more differentiation was shown across motor skills (i.e., above average vs. lowest motor skills). Overall, the pattern of findings challenge the assumption that motor and EF skills progress in lockstep for all children, but rather support the notion that preschool development is marked by periods of reorganization and uneven growth, where different systems mature at different rates with substantial inter-individual differences (Ben-Sasson and Gill, 2014).

The current finding of an *At-Risk* subgroup with weak motor skills and EF difficulties is consistent with the Houwen et al. (2019) study, which identified a subpopulation of children who demonstrated the poorest performance on task-based motor assessments, alongside increased inhibition and working memory problems on a parent report measure of the BRIEF-P. Differences include a smaller sample size in the prior study, a restricted age range of 3–4-year-olds, and the inclusion of a verbal ability score. Interestingly, the prevalence of membership of the *At-Risk* profile in the Houwen et al. (2019) study at 6.7% was lower than ours at 15%. Comparing the strongest-skilled profiles across the two studies, the *High Motor* (12.6%) and *Highly Skilled* (12.1%) profiles are somewhat consistent with Houwen et al.’s (2019) subgroup of 3–4-year-old children with average motor performance and few parent-reported EF difficulties on the BRIEF-P (43.3%). Importantly, the present study extends the earlier work by including a larger sample size and incorporating a parent-report measure of everyday motor coordination together with a broader representation of EF domains, including emotional control, cognitive flexibility, planning and working memory. It is possible that this approach contributed to the emergence of a more differentiated five-profile solution in the current study compared with the three profiles identified by Houwen et al. (2019). The inclusion of a parent-report measure of everyday motor coordination may have captured a broader and more ecologically valid range of motor skills when compared to performance-based assessments that are more structured and rely on timed responses at one timepoint. This increased sensitivity to variability in daily motor performance may have contributed to a more complex pattern of heterogeneity across motor and EF domains during early childhood in the current study.

Regarding our second objective, the current findings indicated significant differences in internalizing symptoms across the five motor–EF profiles. Specifically, children in our *At-Risk* profile showed higher odds of increased internalizing problem behaviors compared to the *Competent* profile, whereas those in our most Highly Skilled profiles (*High Motor* and *Highly Skilled*) had a higher ratio of children with the lowest internalizing symptoms. These patterns suggest that combined vulnerabilities across both motor and EF domains are associated with the highest internalizing problem behaviors, whereas stronger motor skills may buffer against such difficulties even when EF weaknesses are present—as in the *Poor EF* profile. Conversely, low EF—even alongside stronger motor skills—is associated with moderate vulnerability to internalizing behavioral problems indicating that deficits in either domain may contribute to heightened risk. Together, these findings indicate that difficulties in both motor and EF domains may contribute to heightened risk for internalizing problems in preschoolers, but stronger motor skills might to some extent mitigate the effect of EF difficulties on these emotional symptoms.

Furthermore, this pattern of results aligns more generally with growing evidence suggesting that motor difficulties are linked to secondary stressors (e.g., peer problems) that contribute to increased internalizing symptoms in school-aged children with DCD (Cairney et al., 2013; Mancini et al., 2016; Omer and Leonard, 2021). Importantly, the current findings also extend previous research in older children with DCD aged 8–15 years, where parent reported EF skills mediated the association between motor difficulties and internalizing problem behaviors (Omer and Leonard, 2021). However, the current findings revealed heterogeneity across motor and EF skill attributes in younger TD children without a diagnosis of DCD and suggest that parsing the intrinsic heterogeneity across these domains can reveal a previously undetectable at-risk subgroup of children with more emotional problems.

With the exception of Houwen et al. (2019), there is very limited research on the co-development of motor coordination and EF skills in relation to externalizing problem behaviors. However, the current results also showed that our *Poor EF* profile were characterized by a higher odds of having increased externalizing problems when compared to the *Competent* profile. In contrast, our most Highly Skilled profiles (*High Motor* and *Highly Skilled*) had a higher ratio of children with low levels of externalizing symptoms. While this pattern aligns with previous evidence that better motor performance is associated with lower externalizing behavior in 5-and 6-year-old children (Livesey et al., 2006), it is possible that overall EF difficulties represent a more distinct risk marker for high levels of externalizing behaviors including hyperactivity/inattention and conduct problems. These findings are consistent with meta-analytic evidence showing medium effect sizes for the association between overall EF difficulties and externalizing symptoms in preschool children (Schoemaker et al., 2013), and with evidence that severity of early EF difficulties prospectively predicts externalizing problems in later childhood (Yang et al., 2022). However, our cross-sectional design limits any conclusions about potential causal relationships in selective subgroups of preschool children, and the direction of these effects will need to be determined in future longitudinal studies.

In line with expectations, the current findings indicated sex differences across the motor–EF profiles in preschool aged children. Specifically, the *At-Risk* profile with weak fine and gross motor skills and the poorest EF abilities included a higher ratio of boys and younger children while our *Highly Skilled* profile was characterized by a higher ratio of girls than the *Competent* profile. These findings are consistent with the evidence that boys tend to lag behind girls in motor development especially fine motor skills and socioemotional development during the preschool years (Bando et al., 2024; Camden et al., 2015; Piek et al., 2008), and have greater difficulties with EF skills in early childhood when compared to their female counterparts (Mahone and Wodka, 2008). These gender-based discrepancies may partially explain the increased representation of boys in a subgroup with poor motor and EF skills and highlights the importance of early support for boys to facilitate school readiness. Notably, the *High Motor* profile had a higher odds of being older and having higher maternal education compared to the *Competent* profile. One explanation is that children of higher socioeconomic status might have more access to cognitively stimulating materials and experiences than children of lower socioeconomic backgrounds, thereby affording more opportunities to develop fundamental motor skills through participation in play and other activities within the preschool environment (Bart et al., 2007). Taken together, these findings suggest that there may be some gender- and age-related differences in early motor and EF skills evident in preschool-aged children with implications for early intervention.

The current findings can be interpreted within a dynamic systems framework of development (Thelen, 1992; Spencer et al., 2011), which emphasizes that motor, cognitive, and other behavioral domains are interdependent rather than separate. From this perspective, developmental change emerges through continuous interactions among multiple systems, where progress in one domain, such as motor coordination, can support growth in others, such as emotional and behavioral wellbeing. The distinct motor-EF profiles identified in the present study highlight this dynamic interplay and suggest that early variability in motor competence may shape opportunities for social participation, emotional regulation, and behavioral wellbeing. This interpretation also aligns with the notion of developmental discontinuity, wherein the relationship between domains fluctuates across time and context (Petersen, 2023). For instance, early motor vulnerabilities may disrupt these reciprocal processes and contribute to diverging developmental trajectories. From a dynamic systems perspective, these findings align with the view that early childhood development is multicausal and shaped by bidirectional interactions across domains and between the child and their environment. Such a perspective supports the need for integrated assessment and intervention, targeting both motor and EF skills to promote adaptive functioning during the preschool years, helping to build the foundational skills necessary for a successful transition to school.

The current findings showing an at-risk motor-EF profile in early childhood could have important clinical implications for tailored interventions and early screening assessments. It is possible that these nuanced profiles may enhance our ability to identify which children are most likely to benefit from specific types of movement-based interventions. For example, classroom-based interventions such as the Successful Kinesthetic Instruction for Preschoolers (SKIP) program have demonstrated improvements in both fundamental motor abilities and motor-based EF skills (head toes knees shoulders task) in preschool aged children (Mulvey et al., 2018). Similarly, a cognitively challenging motor skills program (delivered twice a week for 8 weeks) showed significant improvements in motor competence, inhibitory control and early numeracy compared to a waitlist control condition in 3–5 year old preschoolers (Hudson et al., 2021). Hence, these types of movement-based programs might be an effective practice to implement with at-risk children with a combination of poor motor skills and EF difficulties to enhance their skills in both domains and reduce the frequency of behavioral problems. This would be important to foster school readiness skills for the successful transition from preschool to more formal school-based settings.

The findings of the current study can be interpreted within a recent conceptualization of EF based on dynamic systems theory (Perone et al., 2021; Spencer et al., 2011). This view proposes that EF and motor skills consist of multiple components in the control of goal-directed behavior within the demands of the specific context, and that developmental domains interact at multiple levels over developmental time (Spencer et al., 2011; Thelen, 1992). This dynamic systems model of goal-directed behavior is relevant to understanding the current findings of discontinuity of motor and EF domains and relationships to internalizing/externalizing problem behaviors across early childhood and suggest that development is multicausal and operates at multiple, interactive levels (Perone et al., 2021). These perspectives highlight the importance of examining both motor and EF domains in early childhood in relation to flexibly adapting to changing environmental contexts.

## Limitations and future directions

There are several limitations of the current study that warrant consideration. First, the current sample were recruited from predominately Western countries including Australia, the United Kingdom, Canada and the United States, which limits the generalizability of the findings, and may lack inclusion of diverse, low socioeconomic communities. Second, the current study relied solely on parent report across all variables inducing shared method variance across constructs. However, this approach does represent an ecologically-valid and efficient approach to collecting developmental information that reflects likely future real-world screening applications. Third, we acknowledge that the high percentage of children at risk for DCD (56.2%) might not be representative of preshool aged children in the general population (Hudson and Willoughby, 2021), and our online recruitment could have introduced self-selection bias in attracting parents who already had underlying concerns about their child’s motor development. Fourth, the Little DCDQ-CA is designed for children aged 36–59 months; however, our age range of 30–69 months could have introduced floor or ceiling effects in fine and gross motor scores that cannot be ruled out. Finally, the cross-sectional design limits any conclusions about how motor-EF profiles change over time, and extent to which profile membership is associated with later internalizing or externalizing problem behaviors and/or diagnosis of a developmental disorder such as DCD.

Notwithstanding these limitations, the current study makes a novel contribution by employing a person-centered approach with the largest known sample to date to delineate motor-EF profiles in the preschool age period, and for the first known time, to link these with internalizing and externalizing behavioral problems. The parent report measures used in the current study avoided the pitfalls of performance-based measures such as participant fatigue, lack of attention, or difficulty in performance under timed conditions, which can impact the validity of assessments and neglect the assessment of real-world manifestations of motor skills and EF in young children.

There are several avenues for future research that could be considered. First, although meaningful subgroups of children were identified based on motor and EF abilities, future longitudinal studies are needed to validate patterns of development across the profile groups over time, and their associations with long-term outcomes or risk for developmental disorders such as DCD and ADHD. Second, it might be a fruitful area of future research to incorporate both parent-rated measures and performance-based assessments and address methodological limitations such as task impurity and factor structure for measurement of EF, and explore the extent to which these types of measures provide complementary information. Third, one area of future research would be to expand on other distal outcomes by including direct measures of autistic/ADHD traits and verbal ability, and determine how these domains vary across profile membership, and the extent to which these profiles are predictive of future likelihood of a developmental disorder. A final avenue of future research would be to include measures of school readiness to determine how these profiles groups may be used to predict children’s preparedness and transition to school. In doing so, researchers may be provided with valuable insights that can be utilized in the implementation of targeted interventions aimed at improving school readiness, especially for children who are identified as at-risk of developmental problems, and thus scaffolding their overall adjustment to formal schooling and promoting long term academic success.

## Conclusion

The findings of the current study provide further evidence for the value of a person-centered approach in capturing the heterogeneity of everyday motor skills and EF in TD preschool children. Using LPA, profiles of motor skills and EF could be derived as representing distinct subgroups with differential relations across these two domains, and highlights the complex interrelationships between these developmental domains. Importantly, two distinct subgroups with elevated risks for behavior problems were identified. The profile with the poorest motor skills combined with EF difficulties had increased odds of elevated internalizing problems, while the profile with EF difficulties but above average motor skills had increased odds of externalizing problems. Taken together, findings suggest that EF difficulties may present a more distinct marker for elevated risk of externalizing behavior problems while a constellation of difficulties in both motor and EF development may increase risk for internalizing problems. These results underscore the importance of recognizing developmental discontinuity and shared weaknesses across motor and EF domains when identifying children for early intervention, and designing early movement-based interventions. By acknowledging the heterogeneity across motor and EF skills, these findings could inform tailored interventions to strengthen foundational skills that transfer across related areas of development and facilitate a smoother transition into formal schooling.

## Data Availability

The raw data supporting the conclusions of this article will be made available by the authors, without undue reservation.

## Footnotes

1
https://www.prolific.com/
